# Single-cell N^6^-methyladenosine-related genes function within the tumor microenvironment to affect the prognosis and treatment sensitivity in patients with gastric cancer

**DOI:** 10.1186/s12935-024-03227-2

**Published:** 2024-01-25

**Authors:** Zehua Wang, Chen Chen, Jiao Shu, Jiaoyu Ai, Yihan Liu, Haoyue Cao, Yongxu Jia, Yanru Qin

**Affiliations:** 1 Department of Oncology, The First Affiliated Hospital of Zhengzhou University, Zhengzhou, 450052 China; 2 Department of Pathology, The First Affiliated Hospital of Zhengzhou University, Zhengzhou, 450052 China; 3 The First Affiliated Hospital of Nanchang University, Nanchang, 330006 China

**Keywords:** Gastric cancer, Single-cell analysis, m^6^A, Tumor microenvironment, Prognosis

## Abstract

**Background:**

Gastric cancer (GC) ranks fifth for morbidity and third for mortality worldwide. The N^6^-methyladenosine (m^6^A) mRNA methylation is crucial in cancer biology and progression. However, the relationship between m^6^A methylation and gastric tumor microenvironment (TME) remains to be elucidated.

**Methods:**

We combined single-cell and bulk transcriptome analyses to explore the roles of m^6^A-related genes (MRG) in gastric TME.

**Results:**

Nine TME cell subtypes were identified from 23 samples. Fibroblasts were further grouped into four subclusters according to different cell markers. M^6^A-mediated fibroblasts may guide extensive intracellular communications in the gastric TME. The m^6^A-related genes score (MRGs) was output based on six differentially expressed single-cell m^6^A-related genes (SCMRDEGs), including GHRL, COL4A1, CAV1, GJA1, TIMP1, and IGFBP3. The protein expression level was assessed by immunohistochemistry. We identified the prognostic value of MRGs and constructed a nomogram model to predict GC patients’ overall survival. MRGs may affect treatment sensitivity in GC patients.

**Conclusion:**

Our study visualized the cellular heterogeneity of TME at the single-cell level, revealed the association between m^6^A mRNA modification and intracellular communication, clarified MRGs as an independent risk factor of prognosis, and provided a reference for follow-up treatment.

**Supplementary Information:**

The online version contains supplementary material available at 10.1186/s12935-024-03227-2.

## Background

Gastric cancer (GC) is the fifth most common malignancy and the third-leading cause of cancer-related mortality globally [1]. Recently, immune checkpoint inhibitors (ICIs) have significantly improved the long-term prognosis in advanced GC patients, especially those with EBV-positive and microsatellite instable (MSI) [2]. However, a large population received limited benefits or develop drug resistance with a median overall survival (OS) of merely 9–10 months [3]. The in-depth understanding of the molecular mechanisms will contribute to bringing new insights for GC treatment.

There has been an increasing interest in post-translational aberration. Among over 150 chemical modifications, N6-methyladenosine (m^6^A) RNA methylation, a new level of epigenetic regulation, is the most abundant modification of protein-coding and non-coding RNAs in eukaryotes [4, 5]. Dynamic m^6^A modification relies on readers, writers, and erasers, respectively responsible for m^6^A’s function, methylation, and demethylation [6]. Roles of m^6^A mRNA regulation on tumor cells have been gradually revealed, modulating tumor progression, stemness, and invasive ability [4]. Recent studies provide evidence of m^6^A mRNA in mediating ICI resistance [7, 8]. It is thereafter that m^6^A modification-related genes may be potential therapeutic targets to optimize immunotherapy.

Tumorigenesis cannot occur without the favor of tumor microenvironment (TME), comprising not just tumor cells but also cancer-associated fibroblasts (CAFs), infiltrating immune cells, vascular cells, mesenchymal cells [9]. Characterization of the cellular composition of the gastric TME is the fundamental to understand immunosuppression, angiogenesis, and distant metastasis [10, 11]. For example, fibroblasts in the TME have been proven to affect the efficacy of ICIs [12]. The high degree of TME heterogeneity represent important barriers to popularizing ICIs. But the cellular milieu contributing to the malignant nature of the gastric TME remains to be elucidated. To date, how m^6^A modification-related genes function within the gastric TME was poorly understood.

Here, we combined single-cell and bulk RNA sequencing to stratify the cellular milieu of the gastric TME, explore how m^6^A-related genes affect intracellular communication in the TME, and screen which m^6^A-related genes are associated with prognosis and guide follow-up treatment in GC.

## Materials and methods

### Data acquisition

52 publicly available human gastric cancer single-cell mRNA sequence (scRNA-seq) datasets (GSE150290) were obtained from the Gene Expression Omnibus (GEO) database (https://www.ncbi.nlm.nih.gov/geo) [13, 14], including 23 tumor tissues and 29 normal adjacent tissues. The bulk mRNA sequence datasets were acquired from The Cancer Genome Atlas program-stomach adenocarcinoma (TCGA-STAD) (https://www.cancer.gov/ccg/research/genome-sequencing/tcga) as a training cohort [15], including 343 tumor samples and 31 normal tissues. The GSE66229 was used as a validation cohort. All data analyzed in our study are freely available in public domain or published literatures.

### Visualization of cell types in the TME

The Seurat R package 4.0.3 was used for processing scRNA-seq data. The quality standards are as follow: (1) each gene expressed in at least three cells; (2) cells with < 200 detected genes were excluded; (3) cells with ≥ 10% mitochondria-expressed genes were excluded. The top 2000 variable genes were used to normalize RNA counts. Principle component analysis (PCA) was performed to identify significant principle components (PCs). Moreover, we used t-distributed stochastic neighbor embedding (t-SNE) algorithm for dimensionality reduction to further obtain the top PCs. Finally, we annotated and visualized the cell types and subtypes constituting the TME of gastric carcinoma.

### Screening single-cell m^6^A-related genes

We used Seurat’s FindAllMarkers to analyze differential expression to get marker genes in each cell type [16]. We downloaded 701 experimentally verified m^6^A-related genes from RM2Target [17]. Then, we obtained single-cell m^6^A-related genes (SCMRGs) by intersecting cell markers and m^6^A-related genes. The expression of SCMRGs was scored using the AUCell package [18].

### Functional enrichment analysis

We performed The Gene Set Variation Analysis (GSVA) to calculate the enrichment score in each cell type. The gene set with a *P*-value < 0.05 was considered significantly enriched. The clusterProfile package was used to conduct Gene Oncology (GO) [19] and Kyoto Encyclopedia of Genes and Genomes (KEGG) analyses [20].

### Pseudotime trajectory analysis

Monocle 2 R package revealed pseudo-time trajectories of fibroblasts [21]. We used the DDRTree method for dimensionality reduction, projecting single cells onto space and ordering into a trajectory with branch points. The dynamic expression heatmap was performed with “plot-pseudo-time-heatmap”.

### Cell-to-cell communication

According to the ligand-receptor interaction database, CellChat can analyze intracellular communication networks of scRNA-seq data annotated as different cell clusters [22]. The number and strength of ligand-receptor interactions were calculated. The “netVisual-circle function” is used to visualize the intensity and number of interactions between cell types, and the “netVisual-bubble function” is used to visualize receptor-ligand pairs between cell clusters. Ligand-receptor pairs with a *P*-value < 0.05 were filtered to explore the interaction between different cell types.

### Identification of SCMRDEGs

DESeq2 package [23] was used to analyze differentially expressed genes (DEGs) between gastric tumor tissues and para-cancer tissues. Significant DEGs were identified by the ‘limma’ package with | logFC | > 1 and adjusted *P*-value < 0.05. The intersection of SCMRG and bulk RNA-seq DEGs was used to obtain differentially expressed single-cell m^6^A-related genes (SCMRDEGs). In the TCGA-STAD cohort, “ConsensusClusterPlus” R package was conducted to cluster GC patients into three clusters based on the expression of SCMRDEGs [24]. The optimal cluster number k = 3 was selected based on cumulative distribution function (CDF). The expression of SCMRDEGs and immune cell infiltration in 3 clusters were compared.

### Establishment and validation of the nomogram model

We used univariate Cox and LASSO analyses to screen SCMRDEGs associated with prognosis. An optimal model was constructed with 6 SCMRDEGs, output as m^6^A-related gene score (MRGs).

$$MRGs=\text{coefficient}+{\sum }_{i}^{1}\beta i\text{*expG}i$$

We used the “survivalROC” R package to verify the predictive efficacy of MRGs in training and validation cohorts, displayed with Kaplan-Meier (K-M) and receiver operating characteristics (ROC) curves [25]. Subsequently, based on univariate and multivariate Cox analyses, we integrated MRGs and classical parameters to establish a nomogram model. Calibration curves were employed to validate the stability of the model.

### Immunohistochemistry

A total of 37 pairs of GC and para-cancerous tissue samples were collected from patients who underwent surgical excision at the First Affiliated Hospital of Zhengzhou University and were used for immunohistochemistry (IHC) between September 2016 and April 2017 [26]. Briefly, IHC assays were performed to assess the expression level of selected SCMRDEGs (GHRL, COL4A1, CAV1, GJA1, TIMP1, and IGFBP3). 4 μm thick paraffin-embedded tissues sections were stained with the GHRL (YT1900, 1:200, Immunoway, USA), COL4A1 (CY1657, 1:150, abways, China), CAV1 (CY5021, 1:150, abways, China), GJA1 (26980-1-AP, 1:200, proteintech, China), TIMP1 (CY6712, 1:150, abways, China), and IGFBP3 (10189-2-AP, 1:200, proteintech, China) antibody per manufacturer’s instructions. The tissue sample collection was approved by the Ethics Committee of Scientific Research of the First Affiliated Hospital of Zhengzhou University. All patients were informed and have written the informed consents.

### Immune infiltration analysis

The MRG-score was divided into high MRGs type and low MRGs type according to the median MRG-score in the training cohort. To assess the relationship between MRGs and immunological state in the TME, we estimated the “StromalScore, ImmuneScore, EstimateScore, and TumorPurity” using the “ESTIMATE” functions of “IOB” R package. The proportional infiltration levels of 22 types of immune cells in two MRGs types were quantified and compared using “CIBERSORT” [27].

### Chemotherapy sensitivity and immune checkpoint analysis

We used the “oncoPredict” package to analyze different drug sensitivity between high- and low- MRGs groups [28]. Six chemotherapeutic drugs with the most apparent difference in half-maximal concentration (IC50) were selected to display. We also analyzed the correlation between MRG score and immune checkpoints, aiming to select the potential immunotherapy-sensitive populations.

### Statistical analyses

Statistical analyses were conducted using R software (version 4.1.2). K-M curves and log-rank tests were applied for survival analyses. A two-sided *P*-value < 0.05 was considered statistically significant.

## Results

### Visualization of TME cell types in GC

**Fig. 1:**
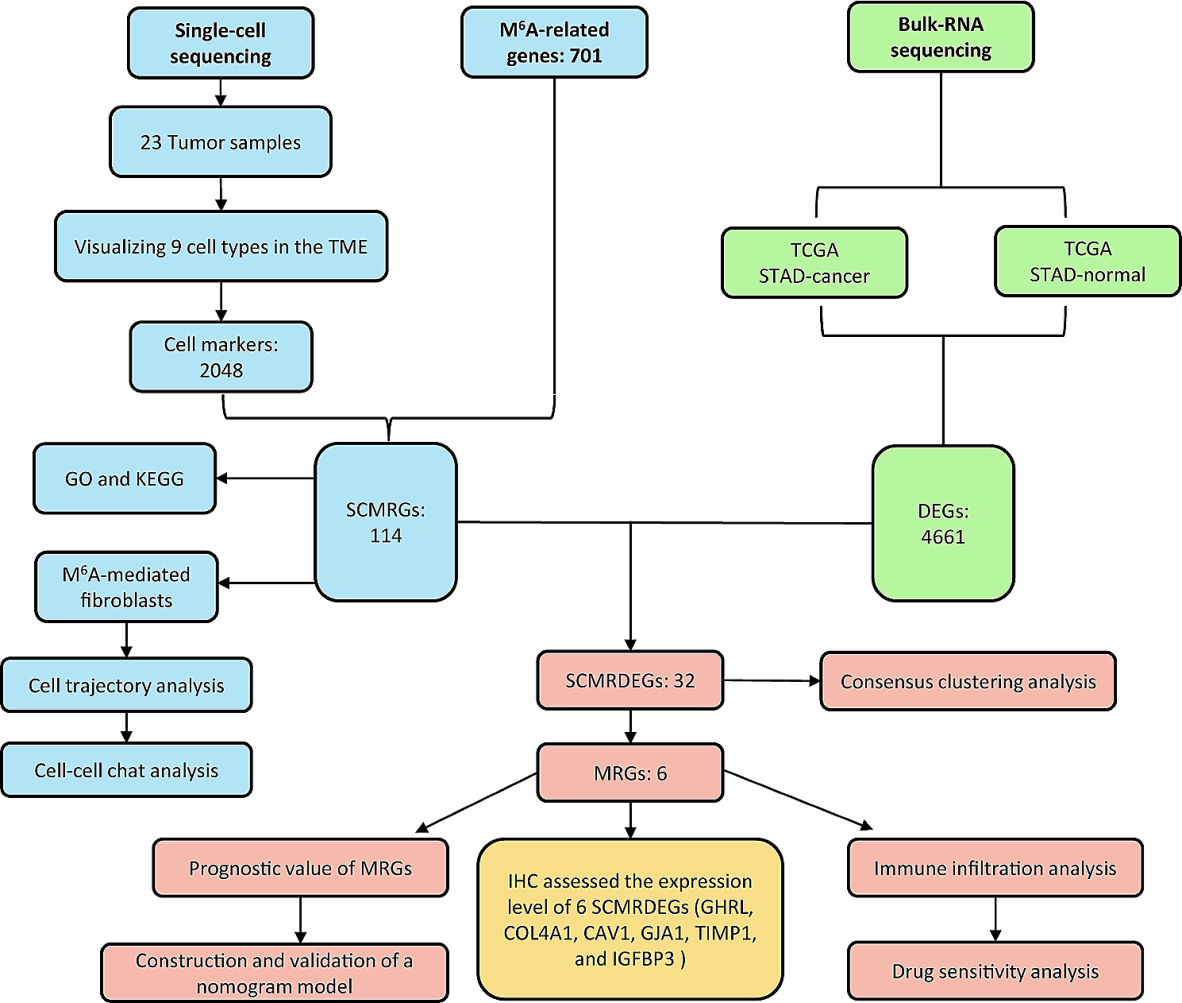
Schematic diagram of the overall study design. SCMRG, single cell m^6^A-related genes; DEGs, differentially expressed genes; SCMRDEGs, single cell m^6^A-related differentially expressed genes; MRGs, m^6^A-related gene score; TME, tumor microenvironment

**Fig. 2:**
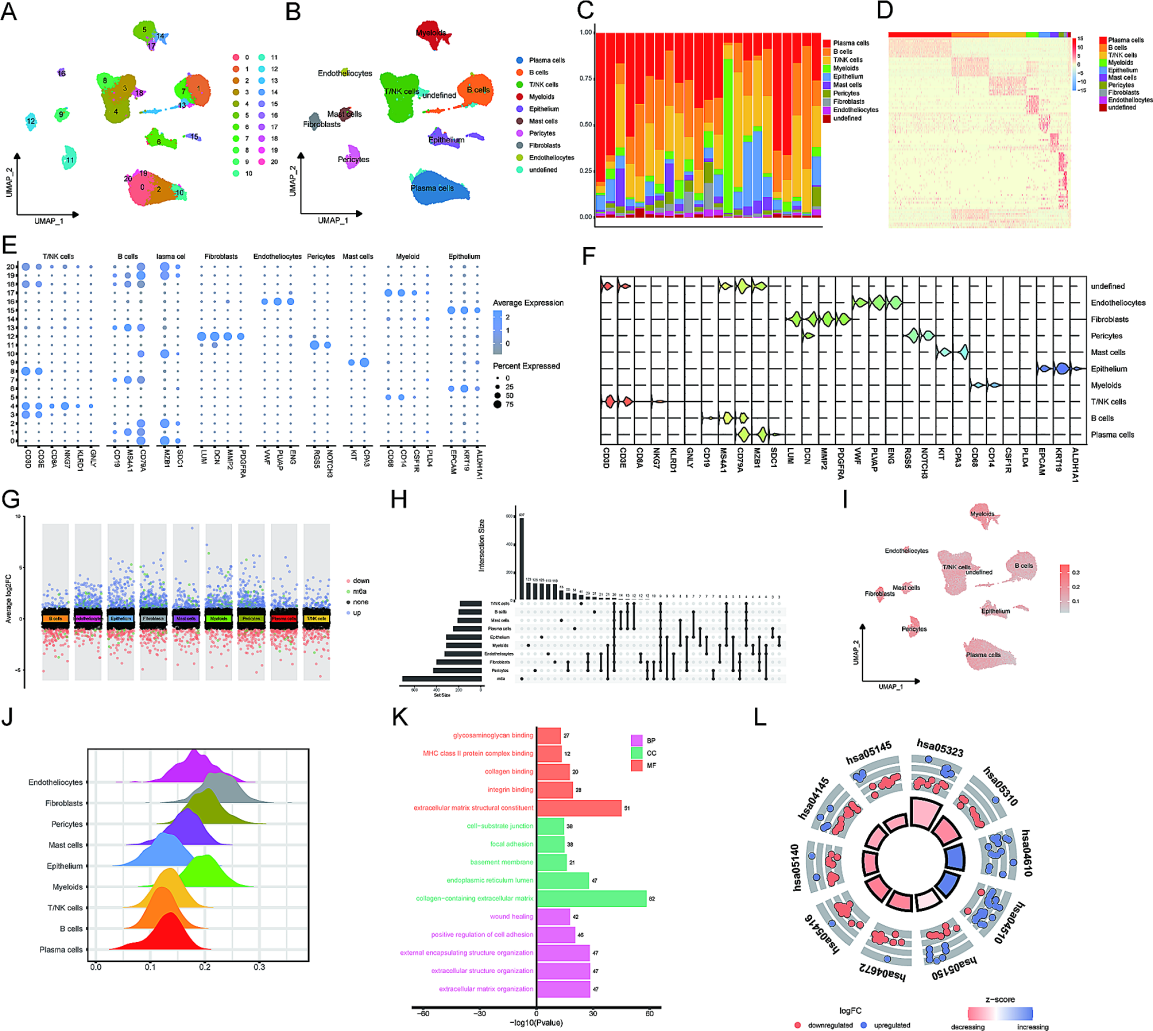
The association of cellular heterogeneity and m^6^A-related genes in gastric TME. **(A)** t-SNE plot of 21 cell clusters. **(B)** t-SNE plot of 9 cell types. **(C)** Distribution of 9 cell types in each included sample. **(D)** Heatmap of highly expressed genes in each cell type. **(E)** Marker genes of each cell type verified by published studies. **(F)** Expression levels of marker genes in the 9 cell types. **(G)** Differentially expressed cell markers in GSE150290. **(H)** Identification of single-cell m^6^A-related genes (SCMRGs); **(I)** t-SNE plot of expression level of SCMRDEGs in each cell type. **(J)** Ridge map of expression level of SCMRDEGs in each cell type. **K.** Gene ontology (GO) analysis of 114 SCMRGs. **L.** Kyoto Encylopedia of Genes and Genomes (KEGG) analysis of 114 SCMRGs.

The overall flow diagram was displayed in Fig. 1. To characterize the TME in gastric cancer, we performed single-cell RNA-seq analysis on 23 tumor samples from the GEO dataset. All cells were divided into 21 clusters (0–20) (Fig. 2A). All clusters could be further divided into 9 cell subtypes, including epithelial cells, endotheliocytes, fibroblasts, myeloid cells, T/NK cells, B cells, plasma cells, pericytes, and mast cells (Fig. 2B). The proportion of each cell lineage in 23 tumor samples was visualized in Fig. 2C. We observed that there are significant differences in the proportion of cell types in different tumor samples, reflecting the high heterogeneity of gastric cancer at the cellular level. The underlying mechanism of heterogeneity might contribute to optimizing individualized treatment. We also plotted different cell types and their corresponding high-expression genes in the heatmap (Fig. 2D). Canonical cell markers identified by previous studies are displayed in Fig. 2E. The cell subtypes we have identified corresponded to their signature cell markers (Fig. 2F).

### Identification of single-cell m^6^A-related genes (SCMRG)

We used the Seurat package to list differentially expressed genes (DEGs) of 9 cell types relative to others, indicating that each cell type has its specific expression profile (Fig. 2G). We identified 114 single-cell m^6^A-related genes (SCMRGs) by intersecting 701 experimentally verified m^6^A-related genes with DEGs (Fig. 2H).

The overall expression level of 114 SCMRGs in each cell type was displayed in a UMAP map (Fig. 2I). The higher SCMRG expression was mainly in the interstitial cells. As shown in the ridge map (Fig. 2J), the expression of SCMRGs was highest in fibrocytes, followed by myeloid cells and pericytes. M^6^A-related genes might play a vital role in the interstitial cells.

To analyze the biological function and associated pathways, we performed GO (Fig. 2K) and KEGG (Fig. 2L) analyses. Regarding biological process (BP), 114 SCMRGs are related to extracellular matrix structural constituent, integrin binding, and glycosaminoglycan binding. For cellular components (CC), these genes are mostly enriched in collagen-containing extracellular matrix and endoplasmic reticulum lumen. For molecular function (MF), these SCMRGs are associated with external encapsulating structure organization, extracellular structure organization, and extracellular matrix organization. KEGG analysis showed that 114 SCMRGs are related to focal adhesion, intestinal immune network for IgA production, and PI3K-AKT signaling pathway.

### M^6^A-mediated fibroblasts guide intracellular communication of the TME

**Fig. 3:**
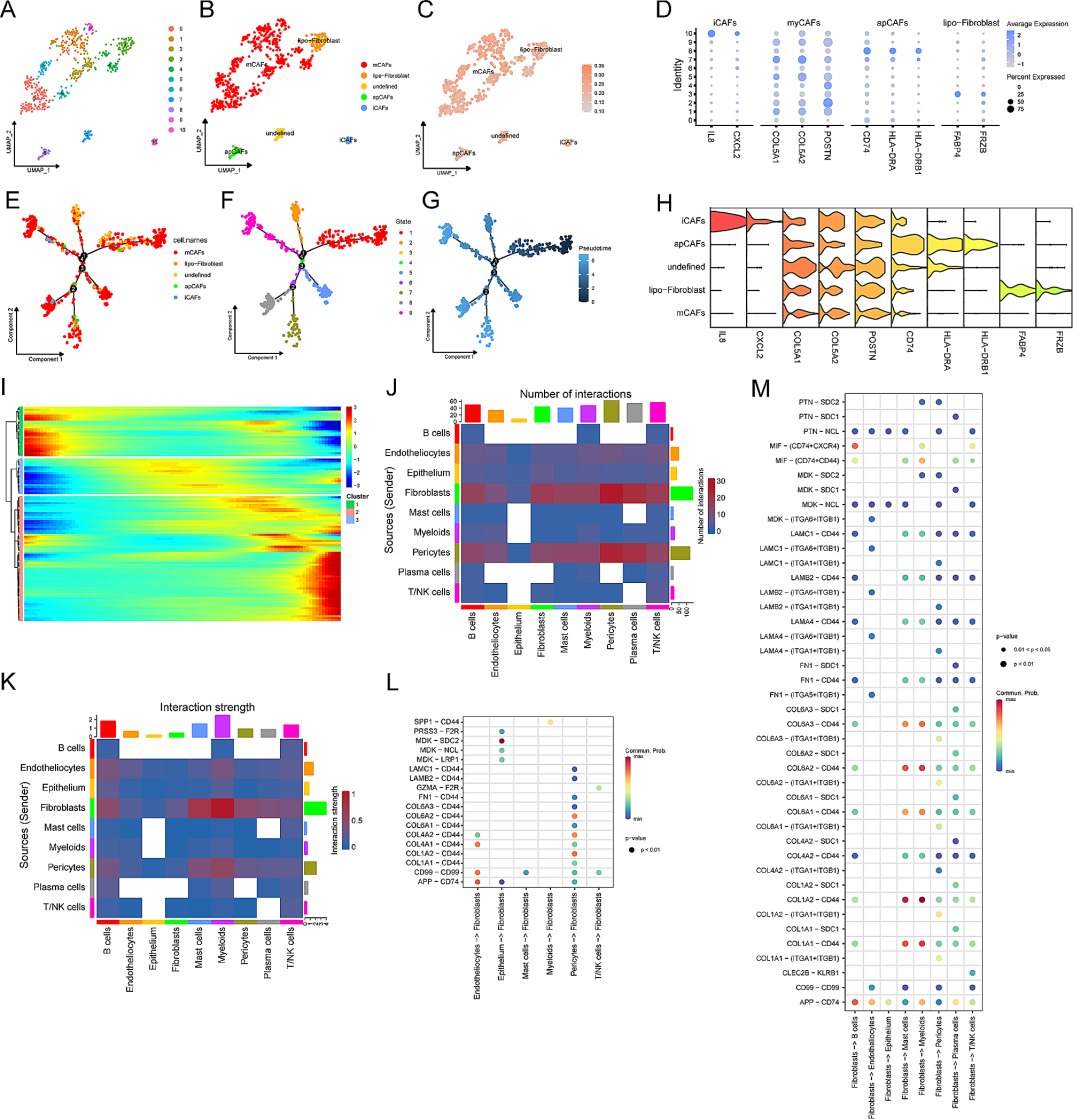
M^6^A-related mRNA modification modifies the features of fibroblasts. **(A)** t-SNE plot of 11 fibroblast subclusters in the TME. **(B)** t-SNE plot of 4 fibroblast subtypes in the TME. **(C)** t-SNE plot of MRG score in 4 fibroblast subtypes. **(D)** Marker genes of each fibroblast subtype. **(E)** Expression level of marker genes in 4 fibroblast subtypes. **F-H.** Differentiation trajectory of fibroblasts, colored for cell types (F), states (G), and pseudotime (H). **I.** Heatmap of dynamic changes of 114 SCMRGs with pseudotime. **J.** The number of interactions among different TME cell subtypes. **K.** The strength of interactions among different TME cell subtypes. **L.** Receptor-ligand pairs that regulate fibroblasts. **M.** Receptor-ligand pairs regulated by fibroblasts

Considering the highest SCMRG expression in fibroblasts, we extracted them for dimensionality reduction clustering. Fibroblasts were divided into 11 sub-clusters (Fig. 3A) and annotated into 4 types: iCAFs, myCAFs, apCAFs, and lipo-fibroblasts (Fig. 3B). There was no difference in SCMRG expression among the four fibrocyte subsets (Fig. 3C). The cell markers of fibroblasts identified by published literature were visualized as a bubble plot (Fig. 3D). The Fibrocyte subsets we identified corresponded to their cell markers (Fig. 3E).

We performed a pseudo-time analysis (Fig. 3F-H) to clarify the differentiation transition of fibrocyte subtypes. These cells formed a continuum with 9 cell states. During pseudo-time, iCAFs and apCAFs gradually decreased while lipo-fibroblast stepwise increased. It is noted that mCAFs did not change significantly. The heatmap (Fig. 3I) showed the 114 SCMRG expression dynamic changes along the pseudo-time. M^6^A-related genes might mediate the differentiation transition among different fibroblast subtypes.

Cell-chat analysis was undertaken to explore diverse interactions between fibroblasts and other cells in the TME. The number and strength of intracellular communication were presented in Fig. 3J and K, respectively. Fibroblasts had a relatively higher intensity and frequency interplay with other cell types. There are the most interactions between fibrocytes and pericytes and the most potent interactions between fibrocytes and myeloid cells. By analyzing receptor-ligand pairs (Fig. 3L-M), fibroblasts mainly serve as signal senders than receivers. Specifically, fibrocytes mainly received regulatory signals from peripheral cells, endothelial cells, and tumor cells. In contrast, fibrocytes could send regulatory signals to regulate all other cell types, consistent with the results in Fig. 3J-K. Therefore, m^6^A-mediated fibroblasts may guide intracellular communications among TME cells, supporting its potential as a novel target.

### Other cell types in the TME

Myeloid cells are crucial components of the TME, exerting both tumor-stimulating and suppressing roles. Subgroup analysis successfully divided 13 distinct clusters (Figure S1A) and revealed 7 myeloid cell subtypes: macro-THBS1, monocytes, macro-APOE, cDC1-CD1C, DC-LAMP3, pDC-LILRA4, cDC1-XCR1 (Figure S1B). We collected specific markers of myeloid cells in Figure S1C. Also, we used a violin plot to validate the expression of typical cell markers in 7 cell subsets (Figure S1D). These markers could well distinguish each myeloid cell subset. We observed that there was no significant difference among the scores of 114 SCMRGs in different myeloid subgroups (Figure S1E).

Although MRG scores of immune cells were not high, T/NK cells were extracted to investigate whether there were differences in MRG scores among different subgroups. Similarly, we further classified 12 cell clusters (Figure S2A) into 5 cell types, including CD4^+^ T cell, CD8^+^ T cell, cycling T cell, regulatory T cell, and NK cell (Figure S2B). T/NK cell markers were collected through single-cell-related studies (Figure S2C). The violin plot was performed to verify the expression level of cell markers in each cell type (Figure S2D). It is noted that there was no significant difference in 114 SCMRG scores among 5 types of T/NK cells from the UMAP plot (Figure S2E).

### Identification of SCMRDEGs

**Fig. 4:**
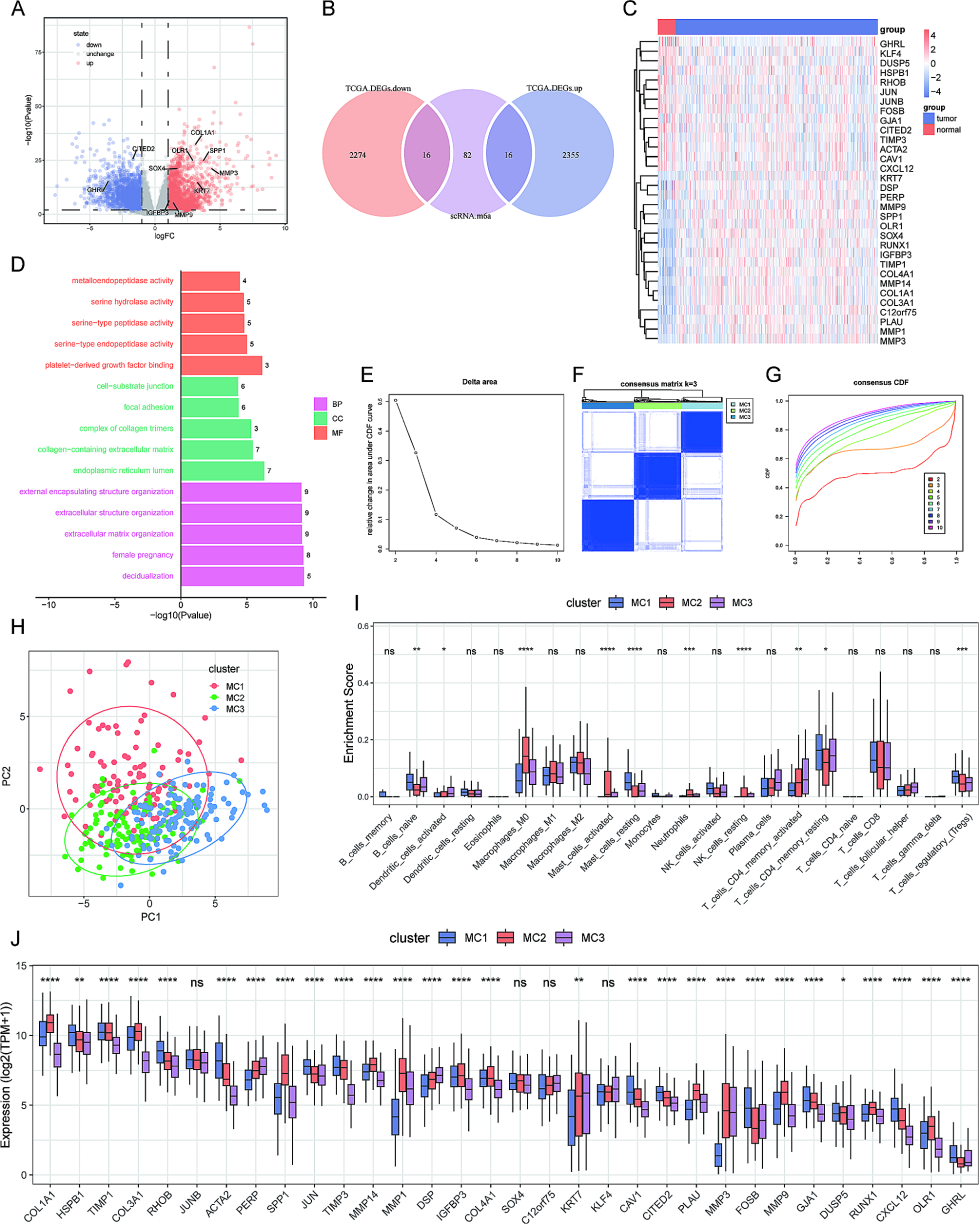
Identification of single cell m^6^A-related differentially expressed genes (SCMRDEGs). **(A)** Volcanic map for differentially expressed genes (DEGs) between normal and tumor tissues. **(B)** Venn diagram indicates overlapped SCMRDEGs between DEGs and single-cell m^6^A-related genes. **(C)** Heatmap of expression levels of DEGs in TCGA-STAD dataset. **(D)** GO analysis of 32 SCMRDEGs. **(E)** Delta area under the cumulative distribution function (CDF) curve. **(F)** Heatmap of clustering at consensus k = 3. **(G)** CDF curves of different consensus k-value. **(H)** Principle component analysis (PCA) of three clusters. **(I)** Immune infiltration of 22 immune cells in the three clusters. **(J)** Differences of immune checkpoints among three clusters

We used the TCGA-STAD transcriptome data to assess differentially expressed genes (DEGs) with the cut-off value of |log2-fold change| ≥2 and adjusted *P*-value < 0.05. A total of 4661 DEGs (2371 upregulated and 2290 downregulated) were identified between normal and tumor samples (Fig. 4A). By intersecting 4661 DEGs and 114 SCMRGs, we finally obtained 32 single-cell m^6^A-related differentially expressed genes (SCMRDEGs), with 16 upregulated and 16 downregulated genes (Fig. 4B). A heatmap (Fig. 4C) showed expression differences of 32 SCMRDEGs between tumor and normal tissues. GHRL, KLF4, and DUSP5 expression levels were relatively lower in tumor tissues, whereas DSP, PERP, and MMP9 were highly expressed in tumor tissues. Furthermore, GO analysis indicated that 32 SCMRDEGs were enriched in metalloendopeptidase activity, serine hydrolase activity, cell-substrate junction, focal adhesion, external encapsulating structure organization, and extracellular structure organization (Fig. 4D).

### Consensus clustering analysis

To clarify the association between SCMRDEGs and GC subtypes, we performed consensus clustering analysis with 343 TCGA-STAD samples. When K > 3, we observed that the area under the cumulative distribution function (CDF) curve did not increase significantly (Fig. 4E). The optimal division was achieved when k = 3 based on the clustering heatmap (Fig. 4F) and consensus CDF (Fig. 4G). Therefore, k = 3 was selected to divide TCGA-STAD samples into three subtypes: MC1, MC2 and MC3. Principle component analysis (PCA) results demonstrated that these 3 samples could not be completely separated by SCMRDEGs but with a clear degree of distinguish (Fig. 4H). The CIBERSORT algorithm compared the immune infiltration score among 3 clusters. As shown in Fig. 4I, Naïve B cells, resting mast cells, resting memory CD4^+^ T cells, and regulatory T cells were enriched in the cluster-MC1; M0-macrophages, activated mast cells, and neutrophils were enriched in the cluster-MC2; activated dendritic cells and activated memory CD4^+^ T cells were enriched in the cluster-MC3. Except for JUNB, SOX4, C12orf75, and KLF4, the expression levels of immune checkpoints were significantly different in all three clusters (Fig. 4J). Notably, the expression level of all immune checkpoints in cluster MC2 were relatively high. It is estimated that patients in cluster MC2 are more likely to respond to immunotherapy.

### Establishment and validation of a nomogram model

**Fig. 5:**
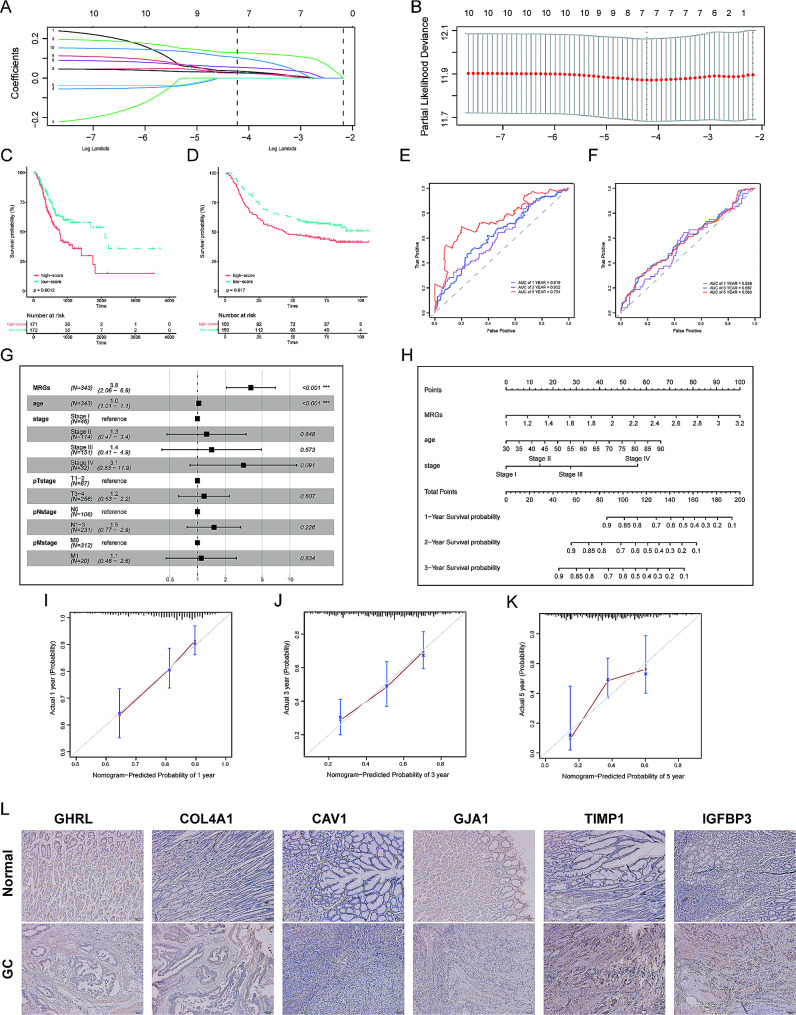
Construction and validation of a MRGs-based nomogram model. **A-B.** The selection of prognostic SCMRDEGs based on the optimal parameter 𝜆 that was obtained in LASSO analysis. **C-D.** K-M survival curves of high-MRGs and low-MRGs groups in training and validation cohorts. **E-F.** ROC curves of MRGs in training and validation cohorts. **G.** Multivariate cox regression analysis in the training cohort. **H.** A nomogram model was constructed with MRGs, age, and AJCC staging. **I-K.** Calibration curves were used to validate the predictive accuracy of our nomogram for predicting 1-, 3-, and 5-year OS. **L.** IHC assessed the protein expression level of GHRL, COL4A1, CAV1, GJA1, TIMP1, and IGFBP3.

Univariate Cox regression analysis screened 10 genes that were associated with the prognosis of GC patients, including COL1A1, TIMP1, COL3A1, ACTA2, TIMP3, IGFBP3, COL4A1, CAV1, GJA1, and GHRL. The selected genes were subsequently included in the Lasso analysis to select the optimal model, which output m^6^A-related gene score (MRGs) based on 6 SCMRDEGs (GHRL, COL4A1, CAV1, GJA1, TIMP1, and IGFBP3) (Fig. 5A-B). The formula is shown as follows:

$$MRGs=\text{coefficient}+{\sum }_{i}^{1}\beta i\text{*expG}i$$

Patients were divided into high and low MRGs groups based on the median value. Kaplan-Meier curves were plotted with THE TCGA-STAD training and GEO validation cohorts. Results showed there were significant prognostic differences (*P* < 0.05) between high- and low-MRGs groups (Fig. 5C-D). The AUCs for predicting 1-, 3-, and 5-year OS in the training cohort were 0.619, 0.632, 0.754 (Fig. 5E), and those in the validation cohort were 0.558, 0.587, 0.583 (Fig. 5F). This finding indicated that MRGs had a favorable predictive ability in the prognosis of GC patients.

Furthermore, the univariate and multivariate Cox regression analyses identified that MRGs and age were independent factors affecting the OS of GC patients (Fig. 5G). AJCC staging also had a significant impact on prognosis. Therefore, a prognostic nomogram model was established based on MRGs, age, and AJCC staging (Fig. 5H). The calibration curves (Fig. 5I-K) of the 1-, 3- 5-year OS demonstrated that the observed results were consistent with the predicted values, verifying the stability of our nomogram model.

The IHC assays (Fig. 5L) verified the differential expressions of six SCMRDEGs in the tumor and adjacent tissues. Of the 24 tissue sections, proteins encoded by COL4A1, IGFBP3, and TIMP1 were slightly up-regulated in tumor tissues, while GHRL and GJA1 proteins were relatively down-regulated in tumor tissues than para-tumor tissues. No significant difference was observed in the expression of CAV1 protein between tumor and normal tissues, partly because it is mainly expressed in the stroma.

### Association of MRGs and immune infiltration

**Fig. 6:**
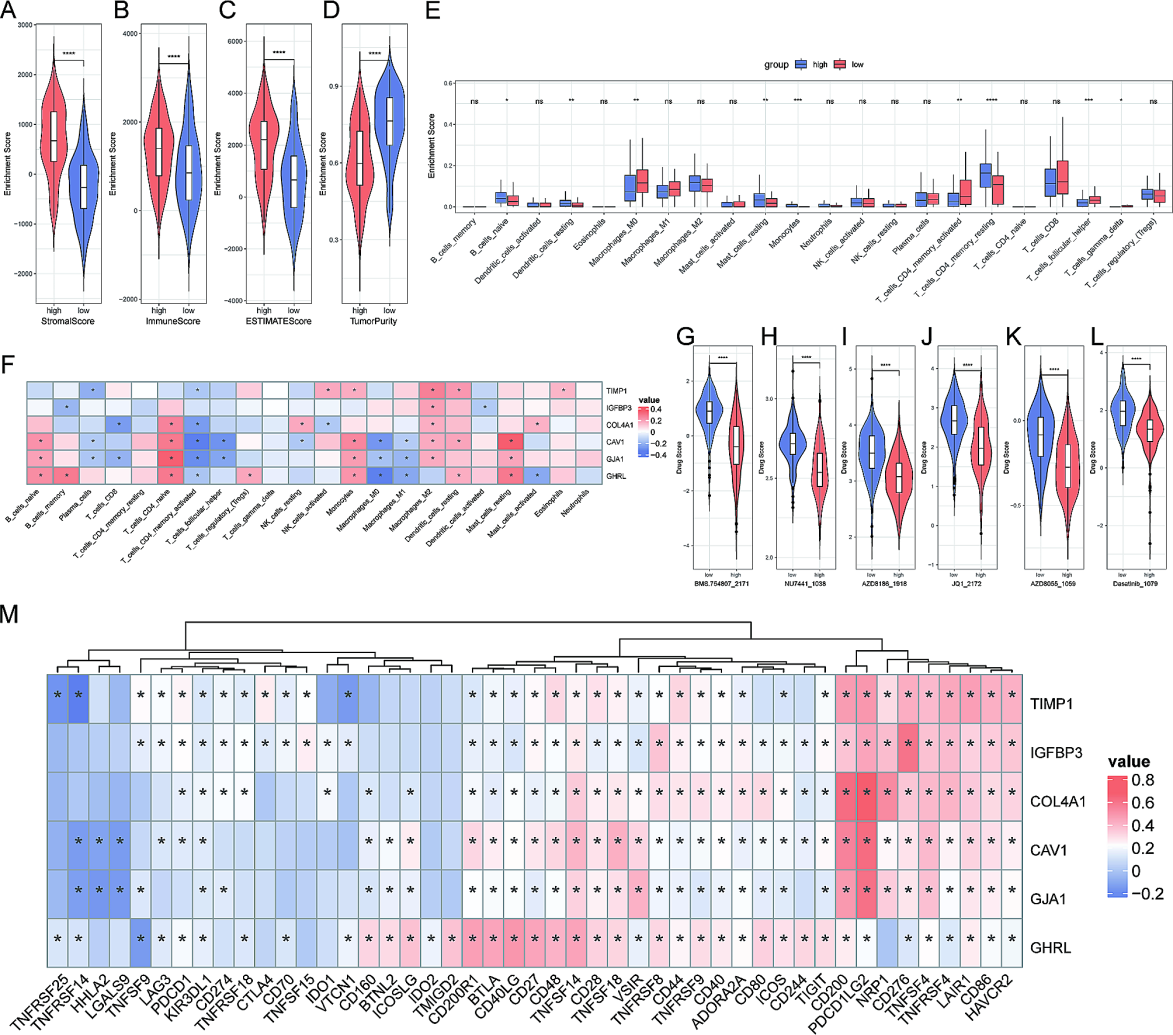
Immune infiltration and drug sensitivity analyses. **A.** Stromal score. **B.** Immune score. **C.** ESTIMATE score. **D**. Tumor purity. **(E)** Immune infiltration of 22 immune cells between high- and low-MRGs groups. **(F)** The correlation between six SCMRDEGs and immune cell infiltration. **G-L.** Violin diagrams of the top six drugs with the most significant difference in drug sensitivity between high-MRGs and low-MRGs groups. **M.** A heatmap displayed the correlations between 47 immune checkpoint molecules and 6 SCMRDEGs.

To infer the relationship between MRGs and immune infiltration, we used the “estimate” R package to calculate Stromal Score (Fig. 6A), Immune Score (Fig. 6B), ESTIMATE Score (Fig. 6C), and Tumor Purity (Fig. 6D). The high-MRGs group had a relatively higher immune score and stromal score compared to low-MRGs group, but the corresponding tumor purity was lower. Moreover, naïve B cells, resting dendritic cells, resting mast cells, monocytes, and resting memory CD4^+^ T cells were enriched in the high-MRGs group, whereas M_0_-macrophages, activated memory CD4^+^ T cells, follicular helper T cells, and γδ T cells had a higher infiltration in the low-MRGs group (Fig. 6E). Also, we found correlations between 6 SCMRDEGs and immune infiltrating cells (Fig. 6F).

### Association of MRGs and drug sensitivity

We used the oncoPredict package to analyze drug sensitivity and obtain the half-maximal inhibitory concentration (IC50) of all chemotherapeutic drugs corresponding to each sample. The violin diagrams (Fig. 6G-L) showed the top 6 drugs with the most significant difference in drug sensitivity between the high-MRGs and the low-MRGs groups. Noteworthily, the high-MRGs group was more sensitive to these 6 chemotherapeutic agents than the low-MRGs group. The heatmap (Fig. 6M) displayed the correlations between 47 immune checkpoints and 6 SCMRDEGs that comprise the MRG score. As the darker color indicates a stronger correlation, TIMP1 was significantly negatively correlated with TNFRSF14 (*P* < 0.05), and GHRL was significantly negatively correlated with TNFSF9 (*P* < 0.05). A significantly positive correlation was observed between IGFBP3 and CD276 (*P* < 0.05). COL4A1, CAV1, and GJA1 were positively correlated with PDCD1LG2 (*P* < 0.05), while both CAV1 and GJA1 were negatively correlated with LGALS9 (*P* < 0.05).

## Discussion

There have been extensive studies on the correlation between m^6^A mRNA modification and the pathogenesis of colorectal cancer [29–33]. Still, fewer studies provided an m^6^A mRNA modification landscape of the GC microenvironment at the single-cell level. In the present study, we leverage the advantage of combined single-cell and bulk RNA sequencing to clarify the heterogeneity of TME in GC, screened significant m^6^A-related genes as independent risk factors, and predicted treatment response in patients with distinct MRGs. This unique perspective may provide a deeper understanding of how m^6^A mRNA modification functions within the TME to impact the prognosis of GC patients.

Recent studies have revealed that the high cellular heterogeneity of the TME supports uncontrolled growth and facilitates immune evasion of solid tumors [34, 35]. In our study, nine cell types were identified preliminarily: epithelial cells, endotheliocytes, fibroblasts, myeloid cells, T/NK cells, B cells, plasma cells, pericytes, and mast cells. We found that cell types with high MRGs are mainly stromal cells. Fibroblasts ranked the highest MRG score, followed by pericytes and myeloid cells. This finding is consistent with subpopulations harboring the highest intensity and frequency of intracellular communications in the TME. It is inferred that m^6^A-related genes may guide extensive and enhanced communications between stromal cells in gastric TME.

Cancer-associated fibroblasts (CAF), a crucial stromal cell component, were further classified as iCAFs, myCAFs, apCAFs, and lipo-fibroblasts, based on specific molecular characteristics [36]. By analyzing receptor-ligand pairs, we found that fibroblasts are more likely to be a sender than a receiver in the cell-chat analysis, sending upstream signals to all other cell types. Also, a recent study manifested that m^6^A-mediated fibroblasts communicate with epithelial cells more extensively than non-m^6^A-mediated fibroblasts in GC [34]. Growing evidence suggested that CAFs may drive an immunosuppressive TME via secreting CXCL2, IL6, and CCL2 [37–40]. Activated fibroblasts are associated with non-response to immunotherapy in pancreatic and breast cancers [41, 42]. It is reasonable to speculate that m^6^A-mediated fibroblasts may form immunosuppressive interplays with other TME cells. Furthermore, m^6^A-related modification might mediate the trajectory process of fibroblast subtypes, regulating various biological processes in GC tumorigenesis and progression. Hence, targeting m^6^A-related fibroblasts is a promising strategy to modulate gastric TME and overcome drug resistance.

We obtained 32 single-cell m^6^A-related differentially expressed genes by integrating bulk RNA sequencing data. Consensus clustering analysis was performed to divide GC samples into three clusters (MC1, MC2, and MC3) with distinct m^6^A expression patterns. We observed that the expression of all immune checkpoints in cluster-MC2 was relatively higher, indicating better immunotherapy responses than other clusters. To further evaluate the predictive value of these SCMRDEGs, we combined univariate and LASSO regression analyses to determine six candidate SCMRDEGs (GHRL, COL4A1, CAV1, GJA1, TIMP1, and IGFBP3) for calculating MRGs. We used the IHC to verify the protein expression level encoded by six SCMRDEGs. Moreover, patients were divided into high- and low-MRGs groups according to the median value of MRGs. By K-M survival and ROC curve analyses, MRGs showed a good predictive ability in the prognosis of GC patients. Patients with high-MRGs have a shorter OS than those in the low-MRGs group. Ultimately, we established and validated a nomogram model based on MRGs, age, and AJCC staging to predict GC patients’ 1-, 3-, and 5-year OS.

Additionally, we assessed the relationship between MRGs and immunological state. Compared with the low-MRGs group, the high-MRGs group tends to have a higher immune and stromal score but lower tumor purity. Multiple resting immune cells showed enrichment in the high-MRGs group, whereas activated immune cells displayed a relatively higher infiltration in the low-MRGs group. Patients in the low-MRGs group may drive a quicker anti-tumor immune response and receive more benefits from immunotherapy. We also comprehensively evaluated the correlations between MRGs and drug sensitivity. Patients in the high-MRGs group were more sensitive to chemotherapy than those in the low-MRGs group. These chemotherapeutic agents include BMS.754807-2172, NU7441-1038, AZD8186-1918, JQ1-2172, AZD8055-1059, and Dasatinib-1079. Therefore, we could use MRGs to predict different responses to immunotherapy and chemotherapy among distinct GC patients.

Some limitations in our study should be highlighted. Firstly, detailed mechanisms of m^6^A mRNA modifications in the multiple TME cells need experimental validation. Secondly, single-cell analysis needs to be more in-depth, and more clinical samples are required. We will verify our findings with a larger sample size and longer follow-up periods in future studies. Thirdly, given that these analyses were based on published databases, it is necessary to validate our findings in real-world cohorts, ensuring the robustness of MRGs as a predictive marker for GC prognosis. Nonetheless, the single-cell and bulk RNA sequencing analyses provide a novel perspective on how m^6^A mRNA modifications function within the heterogenous TME.

## Conclusion

This study integrated scRNA-seq and bulk RNA-seq data to identify m^6^A-modified cellular heterogeneity of TME, reveal m^6^A-mediated fibroblasts guiding intercellular communication of gastric TME, determine the prognostic value of MRGs, and evaluate the effects of MRGs on treatment sensitivity. This study provides a new perspective for an in-depth understanding of the characteristics of m^6^A mRNA modification in the TME cell subtype, which is a critical step for clinical practice and individualized therapy.

### Electronic supplementary material

Below is the link to the electronic supplementary material.

**Supplementary Material 1**: **Fig. S1**. Myeloid cells in the TME. **(A)** t-SNE plot of 13 myeloid cell subclusters in the TME. **(B)** t-SNE plot of 7 myeloid cell subtypes in the TME. **(C)** Marker genes of each myeloid cell type. **(D)** Expression level of marker genes in 7 myeloid cell subtypes. **(E)** t-SNE plot of MRG score in 7 myeloid cell subtypes

**Supplementary Material 2**: **Fig. S2**. T/NK cells in the TME. **(A)** t-SNE plot of 12 T/NK cell subclusters in the TME. **(B)** t-SNE plot of 5 T/NK cell subtypes in the TME. **(C)** Marker genes of each T/NK cell type. **(D)** Expression level of marker genes in 5 T/NK cell subtypes. **(E)** t-SNE plot of MRG score in 5 T/NK cell subtype

## Data Availability

The datasets of this article were generated from the TCGA database and GEO database.
