# Supplementary figures and images for: Single-cell N6-methyladenosine-related genes function within the tumor microenvironment to affect the prognosis and treatment sensitivity in patients with gastric cancer
Source: Cancer Cell Int. 2024 Jan 25;24:44. doi: 10.1186/s12935-024-03227-2 (PMC10811812; doi:10.1186/s12935-024-03227-2)

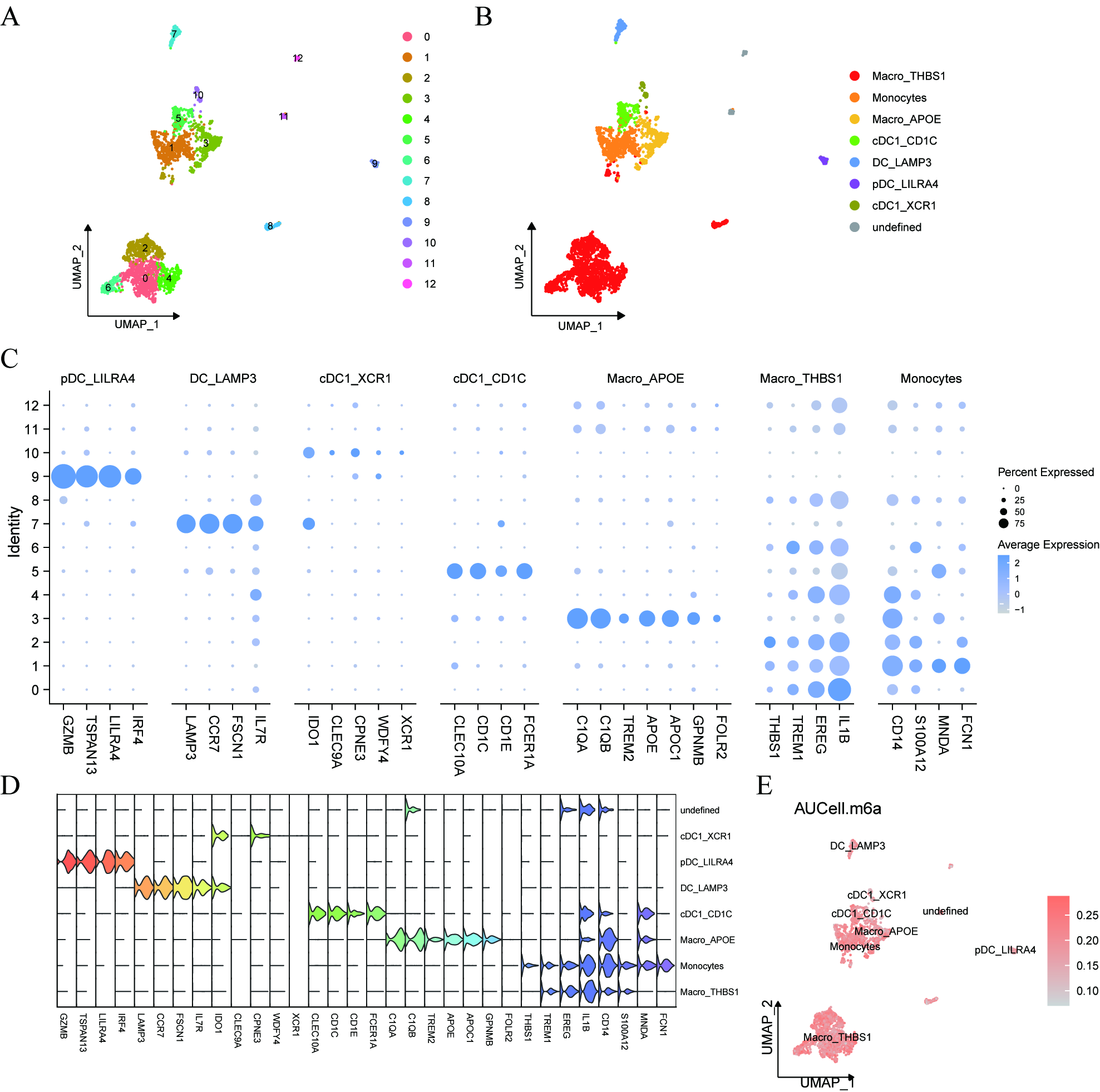

Supplement: Supplementary file 1 — Supplementary Material 1: Fig. S1. Myeloid cells in the TME. (A) t-SNE plot of 13 myeloid cell subclusters in the TME. (B) t-SNE plot of 7 myeloid cell subtypes in the TME. (C) Marker genes of each myeloid cell type. (D) Expression level of marker genes in 7 myeloid cell subtypes. (E) t-SNE plot of MRG score in 7 myeloid cell subtypes [file 12935_2024_3227_MOESM1_ESM.tif]

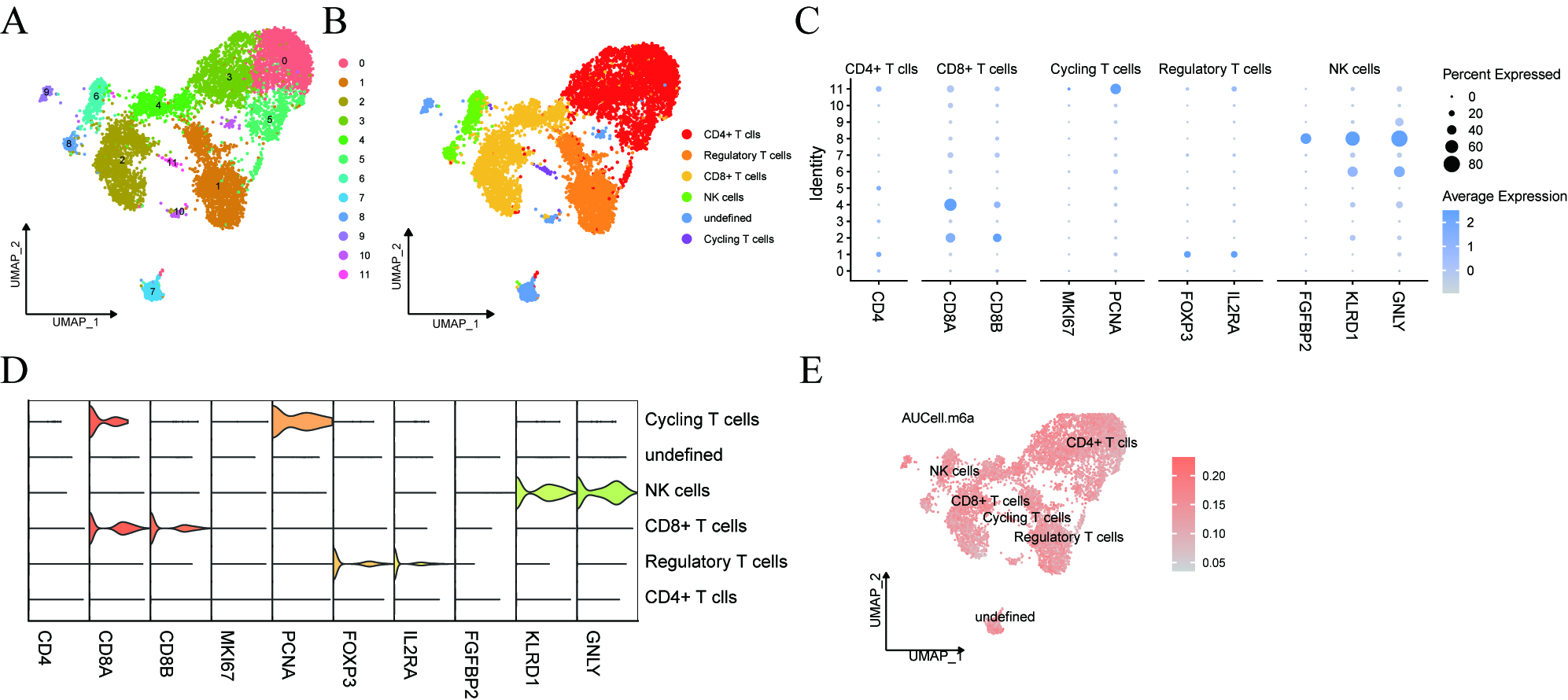

Supplement: Supplementary file 2 — Supplementary Material 2: Fig. S2. T/NK cells in the TME. (A) t-SNE plot of 12 T/NK cell subclusters in the TME. (B) t-SNE plot of 5 T/NK cell subtypes in the TME. (C) Marker genes of each T/NK cell type. (D) Expression level of marker genes in 5 T/NK cell subtypes. (E) t-SNE plot of MRG score in 5 T/NK cell subtype [file 12935_2024_3227_MOESM2_ESM.tif]
